# Nuclear p120 catenin is a component of the perichromosomal layer and coordinates sister chromatid segregation during mitosis in lung cancer cells

**DOI:** 10.1038/s41419-022-04929-z

**Published:** 2022-06-04

**Authors:** Shu-Er Chow, Yaa-Jyuhn J. Meir, Jhy-Ming Li, Ping-Chih Hsu, Cheng-Ta Yang

**Affiliations:** 1 Department of Otolaryngology Head and Neck Surgery, Linkou Chang Gung Memorial Hospital, Taoyuan, 33305 Taiwan; 2 Department of Nature Science, Center for General Studies, Chang Gung University, Taoyuan, 33302 Taiwan; 3 Graduate Institute of Biomedical Sciences, College of Medicine, Chang Gung University, Taoyuan, 33302 Taiwan; 4 Limbal Stem Cell Laboratory, Department of Ophthalmology, Linkou Chang Gung Memorial Hospital, Taoyuan, 33305 Taiwan; 5 Department of Biomedical Sciences, College of Medicine, Chang Gung University, Taoyuan, 33302 Taiwan; 6 Department of Animal Science, National Chiayi University, Chiayi City, 60004 Taiwan; 7 Department of Thoracic Medicine, Linkou Chang Gung Memorial Hospital, Taoyuan, 33305 Taiwan; 8 Department of Thoracic Medicine, Taoyuan Chang Gung Memorial Hospital, Taoyuan, 33378 Taiwan; 9 Department of Respiratory Therapy, College of Medicine, Chang Gung University, Taoyuan, 33302 Taiwan

**Keywords:** Non-small-cell lung cancer, Cell biology

## Abstract

Abnormal expression of p120 catenin is associated with the malignant phenotype in human lung cancer. Numerous studies have focused on the function of p120 catenin in the juxta-membrane compartment. However, the role of nuclear p120 catenin remains unclear. In this study, the dynamic changes in nuclear p120 catenin localization during cell cycle progression were investigated. Immunofluorescent staining, FACS analysis, and western blotting revealed that nuclear p120 catenin is a major architectural constituent of the chromosome periphery during mitosis. During mitosis, granule-like p120 catenin dispersed into a cloudy-like structure and formed cordon-like structures surrounding the condensed chromosomes to create the peri-chromosomal layer. Interestingly, lumican and p120 catenin colocalized at the spindle fiber where the perichromosomal layer connects to the condensed chromosomes during mitosis. Furthermore, downregulation of p120 catenin using a specific siRNA induced cell cycle stalling in the G2/M phase and promoted aneuploidy. This study validates the role of nuclear p120 catenin in the formation of the chromosome periphery and reveals the p120 catenin-lumican interaction may couple orientation of cell division with the segregation of sister chromatids during mitosis. Our data suggest the protective role of p120 catenin in maintaining the integrity of chromosomes, and also warrants further studies to evaluate the contribution of the loss of p120 catenin to the creation of gene rearrangement in cancer evolution and tumor progression.

## Introduction

Lung cancers, the leading cause of cancer-related mortality worldwide, are characterized by extensive genomic alternations within the tumors [1]. Aneuploidy is a crucial, early hallmark of lung carcinogenesis that leads to tumor progression [2]. Although different chromosome imbalances have been related to varied subtypes of lung cancer, variations due to chromosomal instability may result in the transition in tumor morphology and differentiation [3]. The dramatic dissolution of the nucleus during mitosis is accompanied by the assembly of the mitotic apparatus, including nuclear matrix proteins and nucleolar proteins, which are required to facilitate the complicated nuclear division events. Mitotic chromosomes have four structural/functional domains: centromeres, telomeres, the periphery, and arm chromatin [4]. The chromosome periphery (i.e., the perichromosomal layer) may act as a skin that protects the chromosome surface [4], as its components are enriched in ribosomal and nucleolar proteins [4]. Perichromosomal proteins are found along the entire length of chromosomes, excluding the centromeres where sister chromatids are paired and spindle microtubules are connected [5]. Lung carcinoma is highly aneusomic, with gain and loss of entire chromosomes or large chromosome regions [1]. However, little is known about the relationship between aneusomy and perichromosomal protein disposition during mitosis in lung cancer cells.

P120 catenin is a member of the Armadillo gene family that has emerged as a major regulator of cadherin stability and an important modulator of the activity of small GTPases [6]. Similar to the classical cadherins, β-catenin, and α-catenin, p120 catenin plays a crucial role in regulating cell–cell adhesion at adherens junctions (AJs) [7]. P120 catenin recruits the minus ends of microtubules to the cadherin complex to enable the maturation of the junctions. This indicates that p120 catenin may regulate the activity of Rho family GTPases through multiple interactions with their effectors, such as Rho GEFs, GAPs, Rho GTPases, etc. [8–10]. Nuclear p120 catenin signaling is affected by the interaction with Kaiso, which regulates Wnt-responsive genes and transcriptionally represses methylated promoters [11, 12]. Importantly, p120 catenin also acts as a critical regulator of cytokinesis by binding to MKLP1 to spatially control the cycling of RhoA GTPase during cytokinesis [13]. Moreover, abnormal expression of p120 catenin is associated with tumor metastasis and proliferation in lung cancer [6]. Ablation of p120 catenin enhances the invasion and metastasis of human lung cancer cells [13]. Notably, loss of p120 catenin is a driver event of chromosomal instability, suggesting that p120 catenin plays a crucial role in the regulation of cell division [13]. However, the precise function of p120 catenin in mitotic cells remains unclear.

In this study, we explored the mitotic role of nuclear p120 catenin in lung cancer cells. We demonstrate that p120 catenin functions at the chromosome periphery that coats mitotic chromosomes during mitosis. Furthermore, we show nuclear p120 catenin interacts with lumican, a tubulin-binding protein, to coordinate the separation of sister chromatids during the mitotic phase. Depletion of p120 catenin was accompanied by reduced expression of lumican and led to aneuploidy associated with unpredictable chromosome instability. Thus, our data indicate an emergent role for p120 catenin in the modulation of the segregation of sister chromatids during mitosis.

## Materials and methods

### Cell culture

Both non-small lung cancer cell lines used in this study, H460 (HTB-177^TM^) and A549 (CCl-185^TM^), were purchased from the American Type Culture Collection, USA.

### Reagents and antibodies

Unless otherwise noted, all chemical reagents were purchased from Sigma-Aldrich. The antibodies used in the present study were: lumican (ab168348, Abcam), p120 catenin (Novus Technology, Inc. or ECM Biosciences #CM3541), and α-tubulin (Cell Signaling Technology, Inc.). The sequences of the p120 catenin siRNAs target the coding sequence of the gene encoding p120 catenin, *CTNND1* (siRNA 5′-GCUAUGAUGACCUGGAUUA-3′ and 5′-CUAUGAUGACCUGGAUUAU-3′) [14].

### Cell cycle analysis

We adopted the double thymidine synchronization approach to perform cell cycle analysis [15]. Briefly, 25–30% confluent cell cultures were incubated in media containing 2 mM thymidine for 18 h for the first block, rinsed with PBS, incubated in fresh media for 9 h, incubated with 2 mM thymidine for 17 h for the second block to achieve synchronization, and then incubated in fresh media and analyzed by FACS. This protocol enables the collection of cells from the G1/S stage onwards to examine the effect of treatments on cell cycle arrest.

### Confocal immunofluorescent microscopy

To perform confocal microscopy, lung cancer cells were cultivated in chamber slides for 24 h, fixed in 4% formaldehyde at room temperature, rinsed with PBS, then incubated with a combination of anti-lumican/anti-p120 catenin/anti-α-tubulin antibodies at 4 °C overnight. Following 4’,6-diamidino-2-phenylindole (DAPI, Invitrogen) staining for 1 min, the samples were imaged by confocal microscopy (ZEISS LSM 510 META, Carl Zeiss MicroImaging) with the pinhole set at 1 Airy unit under 488, 546, and/or 633 nm excitation using a 63–100/1.4 oil objective lens. The samples were imaged by confocal microscopy (ZEISS LSM 510 META, Carl Zeiss MicroImaging) with the pinhole set at 1 Airy unit under 488, 546, and/or 633 nm excitation using a 63–100/1.4 oil objective lens. To avoid saturation of the fluorescence intensity in the scanned images, we optimized the detector settings using the feature Range Indicator provided by the Zeiss software (Operating Manual LSM510; Zeiss, Jena, Germany).

### Western blotting

Cells were lysed in cold lysis buffer (1% NP-40, 50 mM Tris pH 7.4, 150 mM NaCl, 2 mM EDTA, 50 mM NaF, 10% glycerol) containing Halt Protease and Phosphatase Inhibitor Cocktail (Thermo Scientific), centrifuged at 12,000×*g* for 10 min at 4 ^°^C, and 0.3 mL of the sonicated cell lysates were collected. The Bio-Rad Protein Assay Kit was used to determine the protein concentrations and the cell lysates were resolved by SDS–PAGE and blotted by appropriate antibodies following standard protocols.

### Statistical analysis

All data are presented as mean and standard deviation. We adopted the unpaired Student’s *t*-test to evaluate the significance of the differences between groups. A *p*-value < 0.05 was considered to indicate a significant difference.

## Results

### P120 catenin is associated with cell cycle regulation

**Fig. 1:**
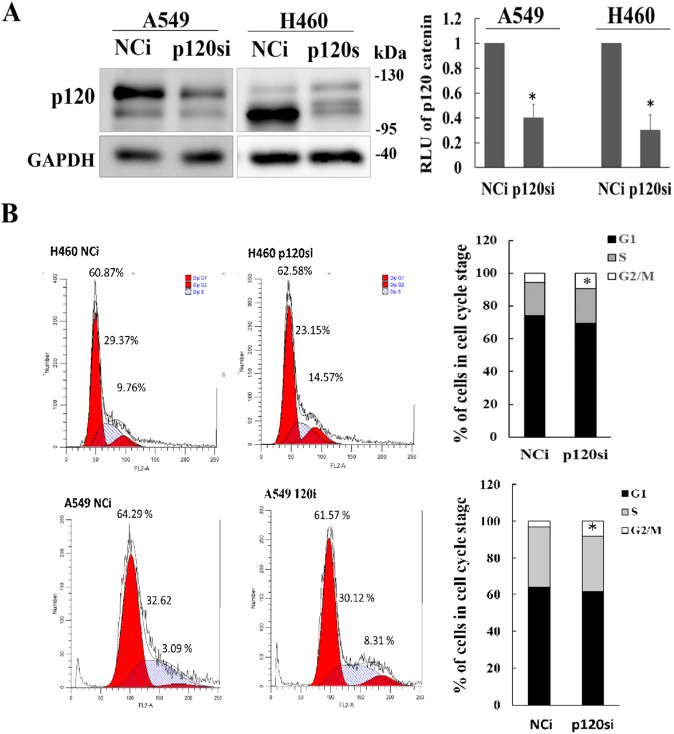
Downregulation of p120 catenin leads to G2/M arrest in H460/A549 cells. Silencing of p120 catenin by transfection of a specific siRNA decreased the expression of p120 catenin. Lung cancer cells H460/A549 were transfected with NCi (negative control siRNA) or p120si (p120 catenin siRNA) and harvested at 24 h. **A** Western blotting of cell lysates with the indicated antibodies confirmed silencing of p120 catenin using p120si effectively decreased the expression of p120 catenin. The expression levels of p120 catenin and GAPDH were determined by densitometry and are expressed in arbitrary units (RLU, relative luminescent units). **B** FACS analysis of the cell cycle profile of transfected cells arrested at the beginning of the S phase using a double thymidine block. Cell synchronization was confirmed by flow cytometry of propidium iodide-stained cells. Representative flow cytometry analysis of the DNA content of NCi/p120si-transfected H460/A549 cells (lower panel). The number of cells (arbitrary units) is plotted against DNA content after release from thymidine block and was quantified using FloJo (right panel); *N* = 3. *p < 0.05 compared with the cells transfected with the control siRNA.

P120 catenin is enriched in lung cancer cells and associated with the stability of cadherin, adhesion-induced signaling, and cell proliferation [6]. To explore the effects of p120 catenin on the cell cycle, we transfected lung cancer cells with negative control siRNA (NCi) or siRNAs targeting the gene that encodes p120 catenin (p120si), and 24 h later, the cells were arrested using a double thymidine block. Silencing of p120 catenin significantly decreased the expression of p120 catenin in A549 and H460 cell lines (Fig. 1A). The cell cycle profiles were monitored by flow cytometry of propidium iodide-stained cells (Fig. 1B). After release from the double thymidine block, the population of NCi-transfected cells at the G2/M phase in H460 or A549 cells was 9.76% and 3.09% (Fig. 1B). In contrast, the population of p120si-transfected H460 or A549 cells retained at the G2/M phase was approximately 14.75% or 8.31%, respectively (Fig. 1B). Thus, this data indicates downregulation of p120 catenin blocked the cell cycle at the G2/M stage.

### P120 catenin is dynamically distributed at the chromosome periphery during mitosis

**Fig. 2:**
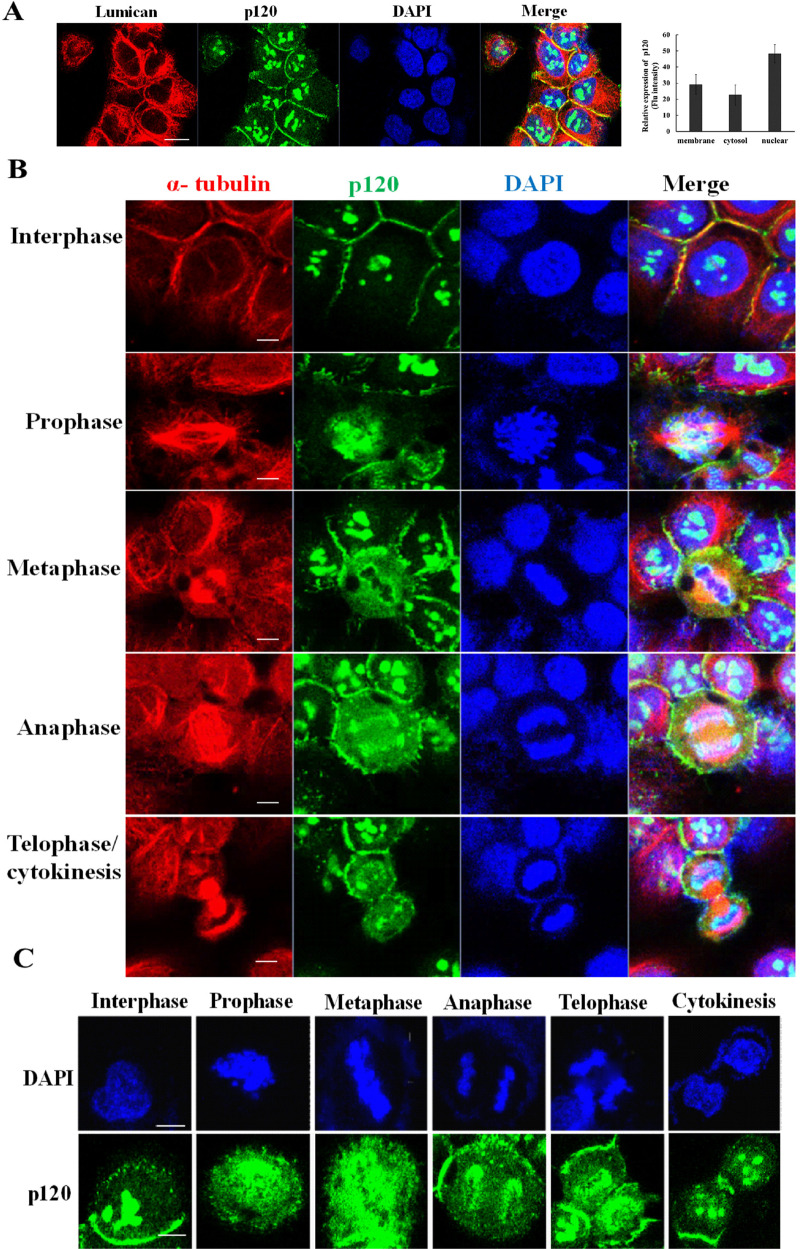
Dynamic morphology of p120 catenin during the processes of cell division. **A** Subcellular distribution of p120 catenin in lung cancer cells. H460 cells were fixed and immunostained using anti-p120 catenin (green) and anti-lumican (red) antibodies. Cell nuclei were visualized by DAPI staining (blue). The arrows indicate the interactions of p120 catenin and lumican at the juxta-membrane region. The relative fluorescence intensity of p120 catenin (green) was quantified using Image J. Scale bar, 20 µm. **B** Distribution and morphology of p120 catenin during cell division. Cells were immunostained using anti-p120 catenin (green) and anti-α-tubulin antibodies (red). DNA was stained with DAPI (blue). Confocal images of cells in interphase, prophase, metaphase, and telophase (cytokinesis) are shown. **C** Dynamic changes in the distribution of p120 catenin were observed during the mitotic stage.

Our previous study has shown the subcellular distribution of p120 catenin investigated by western blot [14]. In addition to the juxta-membrane region, a predominant subcellular distribution of p120 catenin is also observed in the nuclear compartment. To explore the role of the nuclear p120 catenin, the subcellular localization of p120 catenin was verified by immunofluorescent staining with anti-lumican and anti-p120 catenin antibodies in H460 lung cancer cells. Image J was employed to semi-quantitatively assess the fluorescence intensity of p120 catenin (green) in the subcellular compartment. As shown in Fig. 2A, the relative expression levels of p120 catenin in the membrane, cytosol, and nucleus were 29.2 ± 6.2%, 22.6 ± 6.3%, and 48.2 ± 5.6%, respectively. Thus, this data indicates that p120 catenin is abundantly distributed in the nuclear fraction.

The dynamic changes in nuclear p120 catenin observed in mitotic cells in different phases of the cell cycle were investigated by immunostaining with anti-p120 catenin and anti-α-tubulin antibodies (Fig. 2B). At interphase, granule-like p120 catenin (green) staining co-localized with chromatin in the nucleus (DAPI staining). During prophase, p120 catenin appeared to disperse out of the compacted granules observed in interphase and associate with condensing chromosomes (DAPI). Thus, p120 catenin is dynamically relocated into the periphery of the chromosomes. As the cells reached metaphase, p120 catenin formed a cordon‐like apparatus that coated the surface of condensed chromosomes. During anaphase, this cordon‐like apparatus is separated into two parts covering the separated sister chromatids. During telophase/cytokinesis, sister chromosomes are actively tugged toward the opposite poles. In this phase, the cordon-like structures of p120 catenin were disrupted and appeared as granule-like structures accompanying the de-condensed chromosomes. This dynamic distribution and re-aggregation of p120 catenin during mitosis were further validated in A549 cell lines (Supplement Figure). The dynamic behavior of p120 catenin during the four phases of mitotic progression is demonstrated in Fig. 2C. Granule-like aggregation of p120 catenin (green) was observed at the interphase. At the onset of mitosis, these granular structures dissociated into a cordon-like apparatus during prophase/metaphase. Proceeding into anaphase, these cordon-like structures are divided into two cordon-like forms covering the separated chromosomes. During cytokinesis, p120 catenin reorganized into a granular form again. These data indicate that p120 catenin functions at the periphery of chromosomes during mitosis.

### The interaction of p120 catenin with lumican is enriched at the chromosome periphery layer

**Fig. 3:**
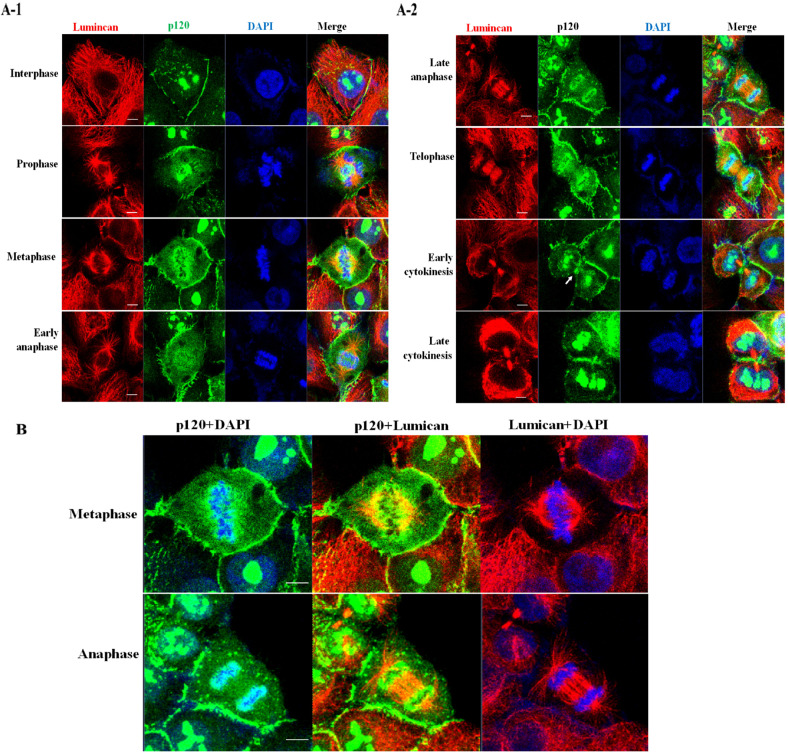
P120 catenin colocalizes with lumican at the boundary of the chromosome periphery. H460 cells were immunostained using anti-lumican (red) and anti-p120 catenin (green) antibodies for 24 h. **A** Confocal images of cells in interphase, prophase, metaphase, and telophase (cytokinesis). **B** High-power merged images of p120 catenin + lumican and p120 catenin + DAPI in the cells in metaphase/anaphase are shown in (**A**).

Lumican, a tubulin-binding protein, is crucial for the formation of spindle fibers and the assembly of midbodies during cell division [15]. Our previous study has shown the interaction of p120 catenin and lumican. The directly physical interaction between lumican and p120 catenin is proved by co-immunoprecipitation and their co-localizations in the juxta-membrane compartment investigated by co-immunofluorescence [14]. To investigate the association of nuclear p120 catenin with lumican during mitosis, we immunostained H460 cells with anti-lumican and anti-p120 catenin antibodies to characterize the dynamic equilibrium of spindle microtubule assembly in the mitotic phase and interphase. As shown in Fig. 3A-1, lumican (red) and p120 catenin (green) colocalized (yellow) at the chromosome periphery during mitosis. During prophase, lumican was present at centrosomes and spindle fibers, and p120 catenin began to dissociate and distribute around the surface of the condensed chromosomes. In metaphase, the chromosomes aligned at the metaphase plate, where p120 catenin formed a cordon-like apparatus enclosing the surface of sister chromatids. Significantly, lumican and p120 catenin colocalized between the boundary of the chromosomes and the spindle fibers (Fig. 3A-1). At the onset of anaphase, the two p120 catenin-mediated cordon-like structures appeared to remain trapped between the sister chromatids, while the two sister chromatids abruptly separated and began moving toward opposite spindle poles. In late anaphase, most of the p120 catenin molecules were clearly observed as cordon-like structures coating the surface of the sister chromatids, as the sister chromatids were actively tugged towards the opposite poles. Colocalization of p120 catenin with lumican was also observed at the mid-plate (Fig. 3A-2). Lumican (red, spindle fiber) radiating from the two polar centrosomes was linked by p120 catenin (white arrowhead) on the perichromosomal layer (Fig. 3A-2). During the transition from telophase to early cytokinesis, lumican also colocalized with p120 catenin at the midzone and the two arms of the midbody. During early cytokinesis, p120 catenin was also present at the cleavage furrow (white arrowhead). In the newly formed nuclei of the two daughter cells, p120 catenin returned to granule-like aggregates. Enlarged images of the metaphase and anaphase cells in Fig. 3A are shown in Fig. 3B. The merged images (yellow) indicated that p120 catenin is a component of the chromosome periphery and interacts with lumican to mediate spindle fiber formation. These data suggest that colocalization of p120 catenin with lumican may assist with orientation and stabilization of chromosomal attachment on astral spindle microtubules during telophase.

### Depletion of p120 catenin disrupts formation of the perichromosomal layer

**Fig. 4:**
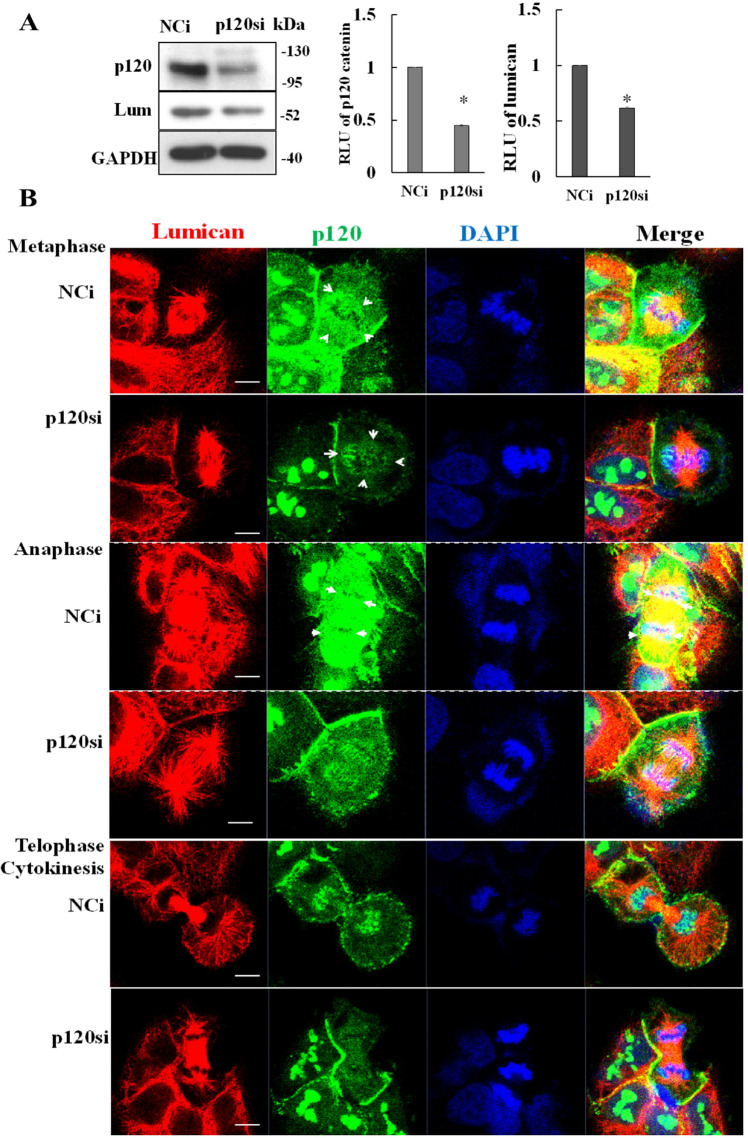
Depletion of p120 catenin decreases formation of the perichromosomal layer. **A** Depletion of p120si effectively decreased the expression of lumican. H460 cells were transfected with p120si and harvested at 24 h. The cell lysates were subjected to western blotting using anti-lumican and anti-p120 catenin antibodies. **B** Depletion of p120 catenin decreased the formation of the perichromosomal layer. The transfected cells were fixed and co-incubated with anti-lumican and anti-p120 catenin antibodies. Lumican (red) and p120 catenin (green) were detected by immunofluorescent staining in H460 cells. Cell nuclei were visualized by DAPI staining (blue). In addition, cells in the metaphase–telophase stage were observed.

To further investigate the role of p120 catenin in the perichromosomal layer, we depleted p120 catenin using specific siRNAs (p120si) and performed immunostaining 24 h later using anti-p120 catenin and anti-lumican antibodies. As shown in Fig. 4A, silencing of p120 catenin effectively decreased the protein levels of both p120 catenin and lumican. To clearly show the relative expression of p120 catenin between NCi- and p120si-transfected cells, we used the intensity of p120si-transfected cells as the detector setting, which resulted in the oversaturated images in the p120 catenin of NCi-transfected cells. The brightness and contrast of the images represented a higher expression in NCi-transfected cells than in p120si-transfected cells. Although the fluorescence signals in the images of Fig. 4B appeared over-saturated, those images did not bias our analysis. Reducing the expression of p120 catenin also impaired the formation of the perichromosomal layer during metaphase to telophase (Fig. 4B). Markedly, the signal for colocalization of p120 catenin with lumican (yellow) at the chromosome periphery reduced after the depletion of p120 catenin. Thus, our data support the notion that the protein levels of p120 catenin play an essential role in the formation of the perichromosomal layer during mitosis.

### Downregulation of p120 catenin increases the incidence of micronuclei and aneuploidy

**Fig. 5:**
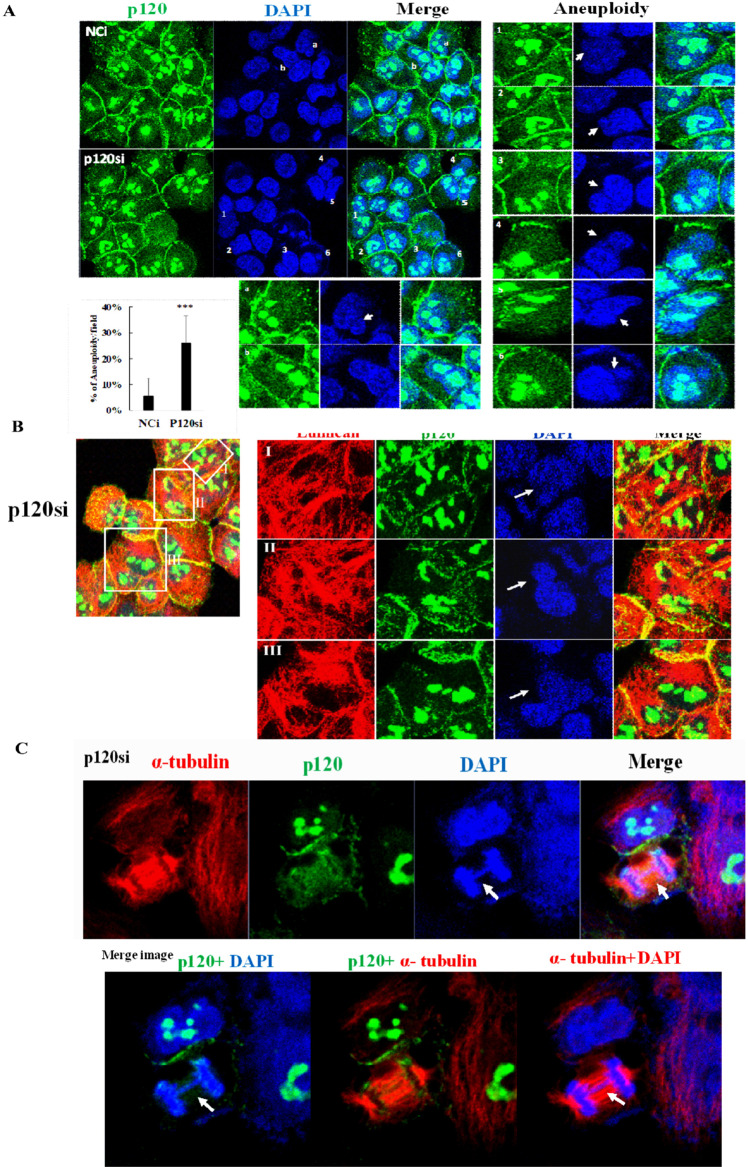
Downregulation of p120 catenin induces aberrant mitosis. P120 siRNA-transfected cells were stained using anti-p120 catenin and anti-lumican antibodies and DAPI to visualize the cell boundary and nuclei, respectively. **A** Multi-nucleated cells immunostained using the anti-p120 catenin antibody (green) and DAPI. Representative images of micro-nuclei/aneuploid cells (marked by the white arrows) in NCi- and p120si-transfected H460 cells. The letters and numbers in the panel represent the images captured in the multi-nucleated cells. Multi-nucleated cells are quantified in the lower panel. **B** Micronuclei were detected in p120si-transfected cells. **C** Immunostaining with anti-p120 (green) and anti-α tubulin (red) antibodies revealed a p120si-transfected cell with a chromatin bridge (indicated by an arrow) during anaphase.

To investigate whether depletion of p120 catenin induced mitotic defects, we visualized the nuclei of the NCi- and p120si-treated cells using DAPI staining. Various sizes of rounded micronuclei were observed in the p120si-transfected cells (Fig. 5A). The number of multi-nucleated cells was about 3.47-fold higher in p120si-transfected cells than in NCi-transfected control cells (13.24% vs. 3.82%, Fig. 5A). Notably, p120si-transfected cells with micronuclei were shown in Fig. 5A and B. Examination of the DAPI-stained nuclei revealed a p120si-transfected cell containing a chromatin bridge, as well as cells with a lower intensity of p120 catenin-contented perichromosomal layer at anaphase (Fig. 5C). These data indicate that the downregulation of p120 catenin increased the incidence of aneuploidy. The presence of cells with micronuclei suggests the occurrence of aberrant chromosome segregation events during mitosis, and that p120 catenin plays an essential role in governing the faithful segregation of chromatids into daughter cells.

## Discussion

P120 catenin engages multifaceted functions associated with its various subcellular distribution [13]. Nuclear p120 catenin abundantly aggregates as irregular, granular-shaped structures during interphase in lung cancer cells [14]. This study reveals that nuclear p120 catenin is a principal architectural constituent of the chromosome periphery by showing that the redistribution and reconfiguration of granulated nuclear p120 catenin are intimately coupled to the cell cycle phases. Thus, p120 catenin may exert a protective role in maintaining chromosomal integrity during mitosis. Furthermore, the interaction of p120 catenin with lumican at the margin of the perichromosomal layer implies that these proteins play a concerted role to ensure proper sister chromatid segregation. Our data suggest that p120 catenin likely acts as a scaffold in the perichromosomal layer to recruit lumican, a spindle fiber component, to ensure mitotic chromosomes are adequately placed onto the spindle apparatus for segregation. This study highlights the role of p120 catenin as a component of the perichromosomal layer that is likely to participate in the regulation of the proper separation of sister chromatids into daughter cells.

### P120 catenin in the perichromosomal layer during mitosis in lung cancer cell lines

Lung cancer is a spectrum of diseases caused by numerous alterations in expression patterns resulting from alterations in various genetic and epigenetic mechanisms [16]. Depending on its cellular localization, p120 catenin can participate in various processes, such as cadherin-dependent cell–cell adhesion, actin cytoskeleton remodeling, and cell proliferation [17]. The armadillo-domain of p120 catenin is first discovered in a complex with Kaiso, and shown to function as a transcriptional repressor. We previously observed increased levels of p120 catenin in the nuclear compartment of A549 and H460 cells [14]. Moreover, in this study, we showed downregulation of p120 catenin-led G2/M phase retardation (Fig. 1). These data strongly suggest that p120 catenin may directly regulate cell division in lung cancer cells.

Targeting of p120 catenin to the perichromosomal layer occurred during prophase to telophase in the processes of mitosis (Figs. 2 and 3). Our p120 catenin knockdown studies demonstrated that p120 catenin functions in the perichromosomal layer, guarding sister chromatids’ integrity during mitotic segregation (Figs. 2 and 3). Since the cells proceed into the mitotic phase, p120 catenin disperses from the irregular granular aggregates and reconstructs into a cordon-like apparatus surrounding the condensed chromosomes during prophase to telophase transit. During cytokinesis and the exit from mitosis, the cordon-like apparatus re-shapes into irregular granule-like structures in the newly formed daughter nuclei (Figs. 2 and 3). These structural shifts indicate that p120 catenin exerts multifaced functions in different mitotic phases; i.e., a perichromosomal layer changes to a cordon-like form that further splits into two coats wrapping around the separated sister chromatids. Thus, downregulating p120 catenin impairs the integrity of the perichromosomal layer and, in turn, leads to aneuploidy (Fig. 4B). As an essential constituent of the perichromosomal layer, nuclear p120 catenin may act as a protective coat to shield the highly organized mitotic chromosomes from their surroundings.

Lumican is overexpressed in lung cancer cells and implicated in tumorigenesis and regulation of cancer cell invasion [14]. Our prior works demonstrated that the physical interaction of p120 catenin and lumican in the juxta-membrane region regulates cell invasion in H460, A549, and other NSCLC cell lines (namely, the H838 and H1957 lines) [14]. The siRNA-mediated silencing of p120 catenin also accompanied a decrease in the expression of lumican [14]. Further, lumican is intensively involved in tubulin-controlled mitotic progression by participating in the formation of centrosomes, mitotic spindle fibers, and the midbody at different stages of mitosis and cytokinesis [15]. The microtubule’s highly dynamic structure represents the target of tubulin-binding anticancer drugs [18]. Its stabilization, resulting in cell apoptosis, is a keystone that underlies drug–drug interaction. For example, paclitaxel interacts with an amino-terminal region of tubulins against depolymerization even at a low concentration [18, 19]. It has served as an anticancer drug in treating non-small cell lung cancer (NSCLC) and other cancers.

Our previous works showed that depleting lumican by its siRNA/sgRNA decreased tubulin’s expression level, which led to aneuploidy/cell apoptosis [15]. Here we showed that depleting p120 catenin also reduced the expression of lumican (Fig. 4A). Thus, manipulating the expression level in either p120 catenin or lumican interferes with mitotic spindle stability and causes chromosomal misalignment at metaphase. The present study provided the first evidence of a mitosis-specific interaction between p120 catenin and lumican that further supports their role in genome integrity maintenance during cell division (Fig. 4), where p120 catenin serves as a surfactant between the chromosomes and spindle fibers, warranting the proper segregation of sister chromatids during mitosis (Figs. 4 and 5). As addressed by the prior studies, one function of p120 catenin in regulating cell cycle progression is related to the cyclin-dependent kinase 2/cyclin E complex [17]. Consistent with this view, the prolongation of the cell cycle in the G2/M phase was observed in the p120 catenin knockdown studies (Fig. 1). Thus, the role of p120 catenin in governing cell cycle progression is evident and consistent with the prior report addressing its relationship with the cyclin-dependent kinase2/cyclin E complex [17].

Our previous works and others showed that p120 catenin is associated with lumican and microtubules to regulate microtubule stability [7, 9]. Therefore, this study provides new mechanistic insight into the pivotal role of the interaction between p120 and lumican in safeguarding chromatid integrity during the nuclear division event of mitosis. Observing p120 catenin colocalizes with lumican and bi-orientally moves toward the cell equator, the cordon-like p120 catenin probably serves as a scaffold in two aspects: (1) to recruit the chromosome–spindle fiber attachment complex for chromatids’ attachment; (2) to warrant proper segregation of sister chromatids as they move towards daughter cells. These observations account for the intricate relationship between p120 catenin, lumican, and tubulin in coordinating spindle fiber formation, which otherwise results in cancer cell dissemination, and poor prognostic outcomes.

Like p120 catenin, other scaffold proteins, such as CUB-domain containing protein 1 (CDCP1), function as a protein–protein interaction hub that interfaces with the signaling proteins and structural elements that also control cell–cell/cell–substratum adhesion, but not the nucleus event of mitosis [7, 20]. Instead, this study unraveled the dynamic distribution of nuclear p120 catenin through a combination effect with lumican explaining how the nuclear p120 catenin hosts these proteins cooperatively to achieve sister chromatid segregation in a high-fidelity manner. Thus, it will be intriguing to identify other players that functioned as p120 catenin, a hub-like complex coordinating between microtubules and the perichromosomal layer, warranting further assessment.

### P120 catenin interacted with lumican in cell cycle

Most solid cancer cells are aneuploid, as they display chromosome number alterations including the loss and gain of complete chromosomes [2]. Chromosomes undergo dynamic structural changes during the transit from interphase to mitosis in eukaryotic cells [4]. p120 catenin is mainly known to control cell–cell adhesion and acts as a critical regulator of cytokinesis [13]. The process of mitotic chromosome assembly is essential for the faithful segregation of duplicated chromatids into daughter cells. Although the loss of p120 catenin induces cytokinesis defects [13] and p120 catenin is associated with microtubules and motor proteins [8], the mechanical details of such functions remain unclear.

Our previous study has confirmed the physical interaction between p120 catenin and lumican and their co-localizations in the juxtamembrane compartment by co-immunoprecipitation and co-immunofluorescence, respectively [14]. The present study further identified the novel role of p120 catenin in the nucleus with a dynamic aggregation and distribution pattern corresponding to chromosomal configuration changes during the transit from interphase to mitosis. As the highly organized mitotic chromosome assembly is essential for the faithful segregation of duplicated chromatids into daughter cells, the periphery domain of the mitotic chromosomes further provides integrity assurance. The periphery of mitotic chromosomes is the domain where specific chromosomal proteins (such as topoisomerase II and K) are involved in maintaining chromosome structure, while others (such as RCC1, Kid, and Kif4) are involved in spindle formation and chromosome movement [21]. Other proteins are also known to localize to the perichromosomal layer of the condensed mitotic chromosomes in mitotic cells. For example, the nuclear protein Ki67, a mitotic chromosome-associated protein, is a component of the perichromosomal layer [22, 23]. However, little is known about the function of the mitotic chromosomal surface. Our present study reports the novel observation that p120 catenin is temporally partitioned to recruit mitotic chromosomes by forming a cordon-like protection shield to promote efficient chromosome separation. Identifying such an unknown function of p120 catenin offers an insight detailing the occurrence of aneuploid in lung cancer cells.

Most importantly, we verified that lumican also plays an essential role in facilitating the faithful sister chromatid’s separation through the physical interaction with the cordon-like p120 catenin. This finding offers a mechanical function of the p120 catenin-mediated cordon-like structure with a mitotic spindle. Consistently, downregulation of p120 catenin decreased the formation of both the perichromosomal layer and spindle fibers due to reduced expression of lumican [14] (Fig. 4). Furthermore, depletion of p120 catenin promoted the generation of aneuploid cells and correlated with multinucleation (Fig. 5), which is recapitulated in the lumican knockdown studies [14, 15]. Therefore, it is likely that the chromosome misalignment observed in p120 catenin-knockout cells may be due to defective spindle organization associated with depletion of lumican. In the same vein, the resulting erroneous attachments led to the formation of lagging chromosomes in anaphase, whereas cytokinesis defects resulted in aneuploid cells (Fig. 5). Thus, our study further identifies p120 catenin as a functional member of the perichromosomal layer and demonstrates that p120 catenin plays a role in mitotic fidelity in combination with lumican.

### Lumican with LRPs provides protein–protein interaction during cell cycle

Lumican belongs to the leucine-rich repeats (LRRs)-containing protein (LRP) [24]. Leucine-rich repeats (LRRs) are 20–29 residue sequence that provides as a protein recognition framework for the formation of protein–protein interaction [25]. Many studies have shown that LRPs interact with diverse cell membrane receptors, cytokines, chemokines, and ECM molecules [25, 26]. Our previous study indicates lumican colocalizes with p120-catenin in the juxta-membrane compartment and is associated with microtubule-modulated p120ctn signaling [14]. A separate study indicates that lumican, a tubulin-binding protein, was associated with microtubule and regulated the expression of tubulin [15]. This study indicated the interaction of p120 catenin with lumican localized in the perichromosomal layer during the mitosis stage. As mitosis approaches, the microtubule-modulated lumican assembles from their interphase arrangement, reorganizes into the spindle structure, and grows until it fills almost the entire cell. Together, these studies might highlight the intricate role of LRP of lumican associated with p120 catenin within various compartments of the cell that affect cell behavior signaling.Like lumican, SHOC2, a tandem array of multiple LRRs-containing proteins, is likely to form a horseshoe-shaped solenoid structure, offering a platform for holding several binding partners and controlling ERK1/2 activity [27]. However, as SHOC2 and other classic LAP (leucine-rich repeats and PDZ domains) proteins with similar LRR structural features [28], whether they interact with p120 catenin is still needed to be investigated.

### Depletion of p120 catenin induced the micronuclei formation and increases the incidence of aneuploidy

Micronuclei arose from lagging chromosomes or chromosome fragments caused by mitotic errors or DNA damage [29]. Delayed chromosome alignment to the spindle equator increases the rate of chromosome missegregation [30]. Chromosome bridges can then undergo cycles of instability, including the formation of new micronuclei [29]. Cells lacking p120 catenin stalled at the G2/M phase and took a relatively long time to align chromosomes on the spindle fibers (Fig. 1); furthermore, micronuclei formation were detected during interphase (Fig. 5B) and a cell with a chromatin bridge was observed during anaphase (Fig. 5C). Micronuclei may set off a cascade of instability, leading to cycles of micronucleus formation and chromosome bridge formation. Thus, DNA in micronuclei might just be the start of a cascade of genome instability [29]. Depletion of lumican associated with the downregulation of p120 catenin results in chromosome missegregation in lung cancer cell lines [15]. These studies suggested targeting the silencing of lumican and p120 catenin increases cell migration and increased aneuploidy of chromosomes. Accordingly, further studies of the role of micronuclei in disease need to be focused on the behavior of the adaptive system.

The consequences of micronuclei formation on tumor biology are dependent on the frequent and irreversible rupture of their nuclear envelopes, which results in the exposure of their DNA contents to the cytoplasm [29]. Notably, chromosomes from micronuclei often rejoin the other chromosomes in the primary nucleus in subsequent cell divisions, which leads to their stabilization within the genome [31]. This study revealed a specific molecular mechanism of p120 catenin in DNA damage and tumor progression. That suggests that micronuclei might be a source of highly localized chromosomal rearrangements. Furthermore, it is reasonable to speculate that the protective role of p120-catenin in maintaining chromosomal integrity may be lost in cancer evolution and tumor progression. Our data warrant more studies to explore the possible contribution of the loss of p120 catenin to the creation of gene fusions, in particular, the acquired rearrangement develops after targeted therapy.

Chromosomal instability may facilitate tumor plasticity and adaptation to versatile environments through Darwinian selection processes, and positively correlates with poorer patient prognosis [32]. Loss of p120 catenin expression occurs as a joint event with multinucleation in breast cancer that strongly correlates and adverse patient survival outcomes [13]. Therefore, downregulation of p120 catenin may not only increase cell invasion but also reduce mitotic fidelity. Aneuploidy may enhance chromosomal imbalances and has detrimental effects on cellular fitness [33]. In contrast, the changes in chromosome copy number could positively contribute to cancer evolution [33]. This fact may in turn account for the relationship between p120 catenin or lumican expression in cancer and increased invasiveness, elevated cancer dissemination, and poor patient outcome. However, future studies are required to investigate the therapeutic effect of targeting lumican for lung cancer treatment.

Together, this study verified a novel role for p120 catenin in the mitotic chromosome periphery and sister chromatid segregation. P120 catenin may function in the perichromosomal layer as a sheath to promote the efficient separation of condensed chromosomes. Moreover, we demonstrate that the interaction of p120 catenin with lumican regulates the microtubule dynamics of the mitotic spindle during mitosis. These activities affect the segregation of sister chromosomes during mitosis, independently of the cadherin adhesion system. Importantly, this study provides new insight into the role of p120 catenin as a guardian of the segregation of sister chromatids during mitosis. Further studies regarding p120 catenin expression, subcellular localization, and chromosome instability in various cancer cell types are currently under investigation.

## Supplementary information

Original Data File

Supplementary Figure legend

Supplement Figure

aj-checklist

## Data Availability

All data generated or analyzed in this study are included in this paper and can be obtained from the corresponding author according to formal requirement.
