# Supplementary material for: Nuclear p120 catenin is a component of the perichromosomal layer and coordinates sister chromatid segregation during mitosis in lung cancer cells
Source: Cell Death Dis. 2022 Jun 4;13(6):526. doi: 10.1038/s41419-022-04929-z (PMC9167299; doi:10.1038/s41419-022-04929-z)

Fig. 1
Western blot

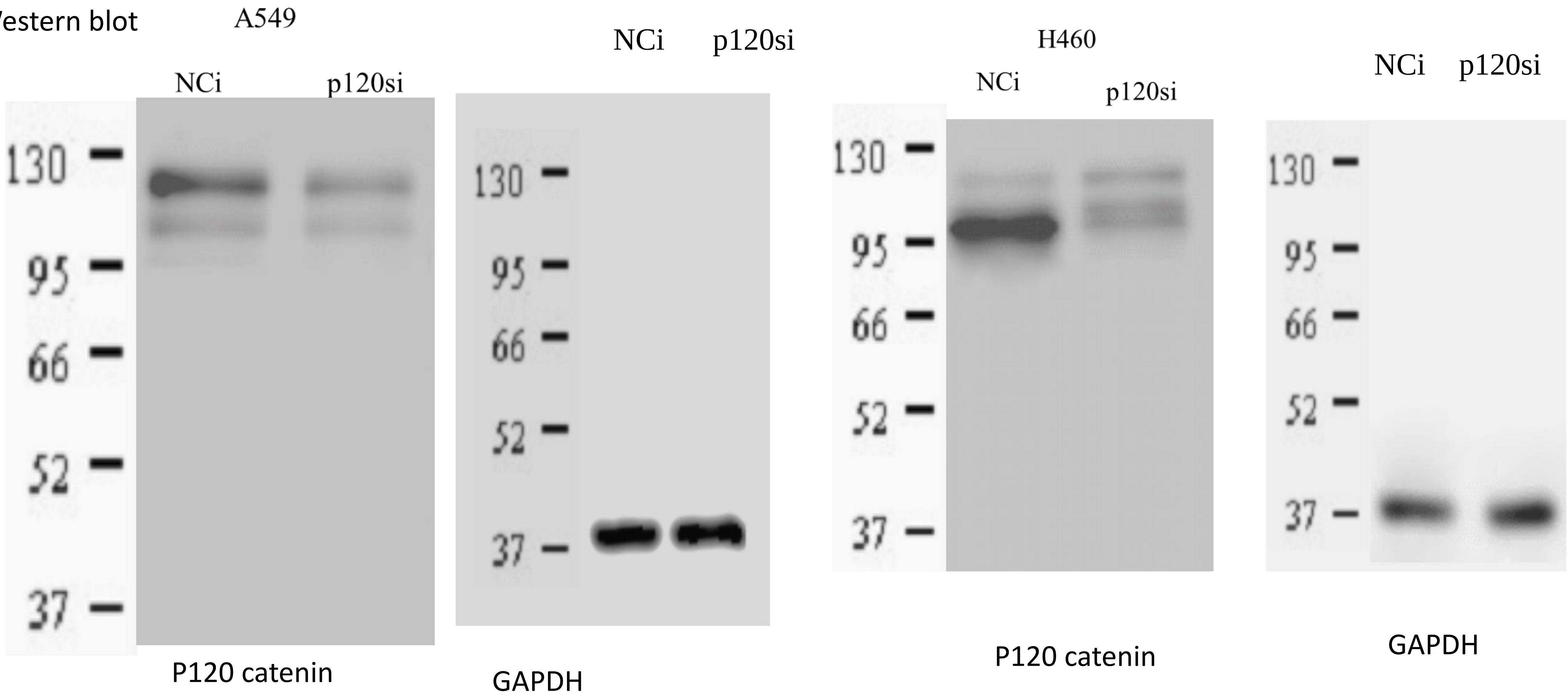

Fig. 4A
Western blot

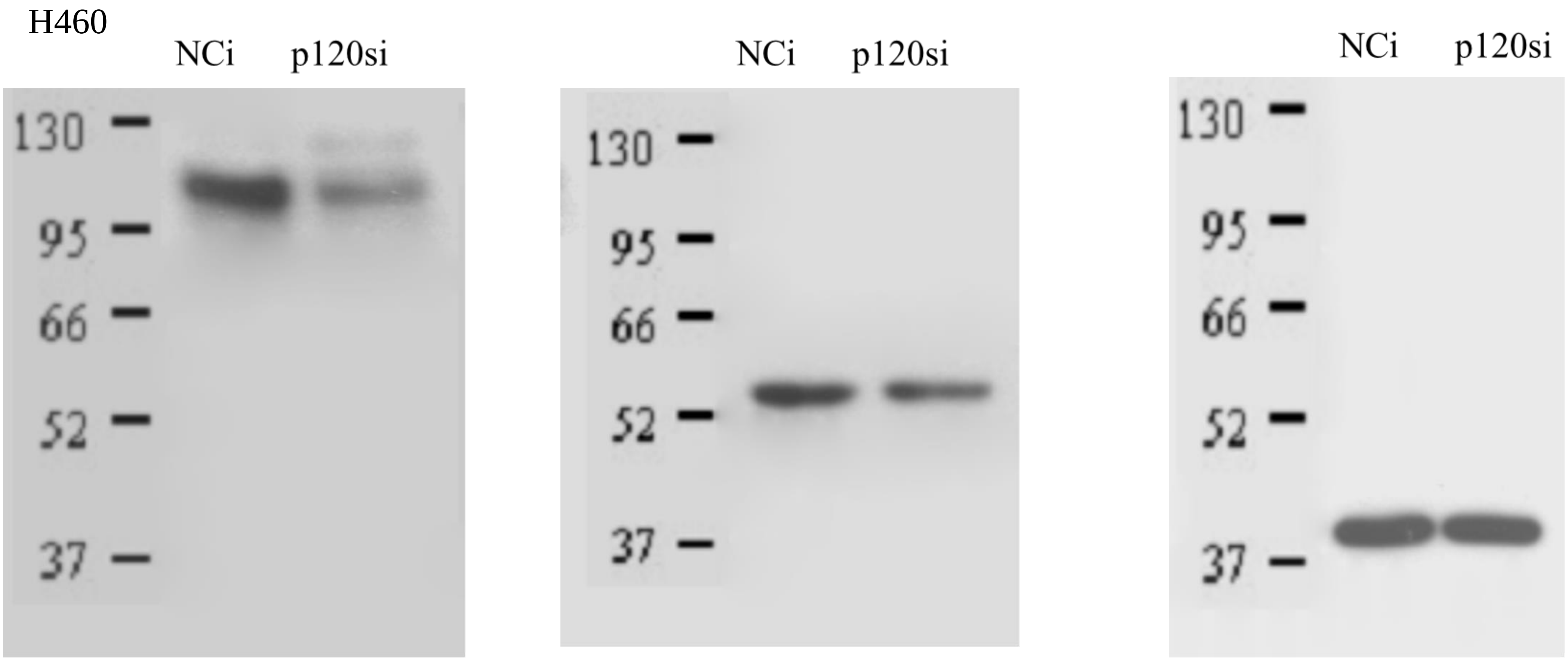

Supplement: Supplementary file 1 — Original Data File [file 41419_2022_4929_MOESM1_ESM.pdf]
