# Supplementary material for: Nuclear p120 catenin is a component of the perichromosomal layer and coordinates sister chromatid segregation during mitosis in lung cancer cells
Source: Cell Death Dis. 2022 Jun 4;13(6):526. doi: 10.1038/s41419-022-04929-z (PMC9167299; doi:10.1038/s41419-022-04929-z)
Supplement: Supplementary file 2 — Supplementary Figure legend [file 41419_2022_4929_MOESM2_ESM.docx]

**Suppl Fig. Dynamic morphology of p120 catenin during the processes of A549 cell division**

Distribution and morphology of p120 catenin during A549 cell division. Cells were immunostained using anti-p120 catenin (green) and anti-α-tubulin antibodies (red). DNA was stained with DAPI (blue). Confocal images of cells in interphase, prophase, metaphase, and telophase (cytokinesis) are shown.
