# Supplementary figures and images for: Nuclear p120 catenin is a component of the perichromosomal layer and coordinates sister chromatid segregation during mitosis in lung cancer cells
Source: Cell Death Dis. 2022 Jun 4;13(6):526. doi: 10.1038/s41419-022-04929-z (PMC9167299; doi:10.1038/s41419-022-04929-z)

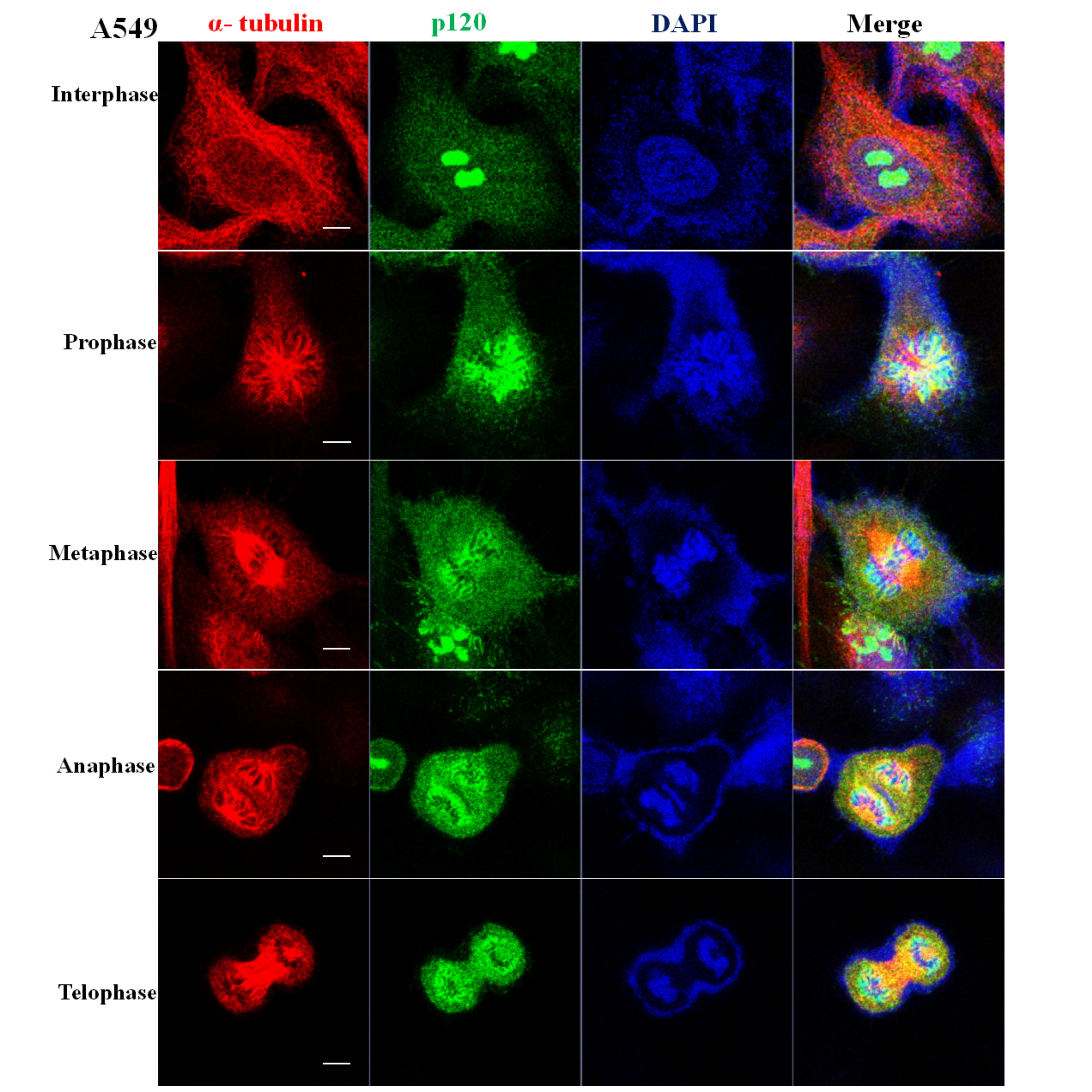

Supplement: Supplementary file 3 — Supplement Figure [file 41419_2022_4929_MOESM3_ESM.jpg]
